# Mitochondrial Dysfunction Induces Formation of Lipid Droplets as a Generalized Response to Stress

**DOI:** 10.1155/2013/327167

**Published:** 2013-09-22

**Authors:** Seon-Jin Lee, Jinglan Zhang, Augustine M. K. Choi, Hong Pyo Kim

**Affiliations:** ^1^Biomedical Genomics Research Center, Korea Research Institute of Bioscience and Biotechnology (KRIBB), Daejeon 305-806, Republic of Korea; ^2^Department of Surgical Intensive Care Unit, Capital Medical University Affiliated Beijing Anzhen Hospital, Beijing Institute of Heart, Lung and Blood Vessel Disease, Beijing 100029, China; ^3^Pulmonary and Critical Care Medicine, Brigham and Women's Hospital, Harvard Medical School, Boston, MA 02115, USA; ^4^School of Pharmacy, Ajou University, Suwon 443-749, Republic of Korea

## Abstract

Lipid droplet (LD) formation is a hallmark of cellular stress. Cells attempt to combat noxious stimuli by switching their metabolism from oxidative phosphorylation to glycolysis, sparing resources in LDs for generating cellular reducing power and for anabolic biosynthesis. Membrane phospholipids are also a source of LDs. To elucidate the formation of LDs, we exposed mice to hyperoxia, hypoxia, myocardial ischemia, and sepsis induced by cecal ligation and puncture (CLP). All the above-mentioned stressors enhanced the formation of LDs, as assessed by transmission electron microscopy, with severe mitochondrial swelling. Disruption of mitochondria by depleting mitochondrial DNA (**ρ**0 cells) significantly augmented the formation of LDs, causing transcriptional activation of fatty acid biosynthesis and metabolic reprogramming to glycolysis. Heme oxygenase (HO)-1 counteracts CLP-mediated septic shock in mouse models. In HO-1-deficient mice, LD formation was not observed upon CLP, but a concomitant decrease in “LD-decorating proteins” was observed, implying a link between LDs and cytoprotective activity. Collectively, LD biogenesis during stress can trigger adaptive LD formation, which is dependent on mitochondrial integrity and HO-1 activity; this may be a cellular survival strategy, apportioning energy-generating substrates to cellular defense.

## 1. Introduction

Intracellular lipid droplets (LDs) store neutral lipids, including triacylglycerol and cholesterol esters, in cells [1]. LDs are surrounded by a unique phospholipid monolayer that is decorated with many proteins, among which adipocyte differentiation-related protein (ADRP) and the perilipin family are well characterized [2–4]. Although LDs were initially considered to be static organelles for fat storage, it is becoming increasingly evident that alterations in the regulation of formation and breakdown of LD can affect the risk of metabolic disorders such as diabetes and related diseases [5–8].

Recently, the biology of LDs has gained public interest because of the causal link between excess lipid storage in certain tissues and pathophysiologies related to obesity [9, 10]. In these cases, LDs are likely to interfere with membrane trafficking of the insulin-sensitive glucose transporter, which might account for the insulin resistance common in patients with type 2 diabetes [11]. 

LDs are formed under two very different environmental conditions that are not mutually exclusive. First, cells accumulate LDs in response to exogenous lipid availability. It is thought that the fatty acids in LDs are stored for energy generation [12], use as membrane building blocks [13], and biosynthesis of steroid hormones [14]. Second, many kinds of cellular stress, including inflammation and oxidative stress, can also induce LD biogenesis [15, 16]. In these cases, it is hypothesized that fatty acids may serve a protective role against the stressors.

The underlying molecular mechanisms of fatty acid uptake and biosynthesis under stress conditions remain largely elusive. Fernàndez et al. demonstrated that the liver of caveolin-1-deficient mice fails to store triacylglycerol in LDs after partial hepatectomy [17]. Caveolin-1 is a structural protein of plasmalemmal caveolae and has a role in lipid homeostasis and caveolae-mediated endocytosis [18, 19]. Caveolin-deficient hepatocytes have few LDs and do not divide for liver regeneration [17]. Administration of glucose to cav1^−/−^ mice increased survival and caused hepatocytes to reenter the cell cycle [20]. The above results imply that caveolin-1 may modulate both lipid metabolism and LD formation [19], providing survival benefit by assisting liver regeneration. 

Heme oxygenase (HO)-1, an inducible stress protein, confers cytoprotection against different oxidative stresses *in vitro* and *in vivo*. In addition to its physiological role in heme degradation, growing evidence demonstrates that HO-1 can influence a number of cellular processes, including growth, inflammation, and apoptosis [21–24]. It is generally accepted that HO-1, a major cytoprotective enzyme, attenuates oxidative stress [25, 26]. Recent observations suggest that HO-1 may modulate obesity and related metabolic disorders, implying a metabolic role for the enzyme.

In the current study, we showed that different kinds of stressors induce LD formation in mouse tissues, with concomitant upregulation of LD-trafficking proteins. Mitochondrial dysfunction may trigger LD biogenesis, which was not observed in the absence of HO-1 activity. Therefore, LD appears to mediate the cytoprotective activity of HO-1 under stress.

## 2. Materials and Methods

### 2.1. Animal Oxygen Exposure

Wild-type C57BL/6 mice (Jackson Laboratories, Bar Harbor, ME, USA), 8–12 weeks old, were maintained in laminar flow cages in a pathogen-free facility. All procedures were performed in accordance with the Council on Animal Care at Harvard University and the National Research Council's Guide for the Humane Care and Use of Laboratory animals. The C57BL/6 mice were exposed to room air or hyperoxia (95% O_2_, 5% N_2_) for up to 72 h. Lung tissue was harvested from the mice at the indicated intervals and used for the biochemical analysis of autophagy [27]. Wild-type C57BL/6 mice were exposed to hypoxia (10% O_2_) or normoxia for 3 weeks, and then their lungs were harvested and analyzed for LD formation by electron microscopic analysis [28, 29]. 

Acute myocardial infarction (AMI) was induced in rats as described previously. In brief, animals were anesthetized by intraperitoneal injection of sodium pentobarbital (50 mg/kg), and AMI was established as described previously [30]. After implanting the electrocardiogram monitor, the rat was connected to a respirator through tracheotomy, and the heart was rapidly exteriorized through left thoracotomy and pericardial incision. The left anterior descending coronary artery was ligated. Successful establishment of AMI was confirmed by the pale appearance of the anterior wall of the left ventricular apex, weakened pulse, and ST-segment elevation. The hearts were harvested 40 minutes, 24 hours after ligation of the coronary artery in the AMI group. The rats in the sham-operated group underwent the same procedure except for the ligation of the coronary artery.

### 2.2. Cecal Ligation and Puncture (CLP) Model of Polymicrobial Sepsis

The CLP model of polymicrobial sepsis was performed as previously described [21]. Briefly, anesthesia was induced in mice by intraperitoneal administration of 100 mg/kg ketamine HCl and 43 mg/kg xylazine HCl. The mouse cecum was exposed through a 1.5 cm incision, the cecum was ligated below the ileocecal valve with a 6–0 silk suture without causing bowel obstruction, and then, the cecum was punctured with a 19-gauge needle. A 19-gauge, 1-hole injury was performed in studies using HO-1-null mice on a BALB/c background. The cecum was repositioned, and the abdominal incision was closed in layers with 6–0 surgical sutures. Sham-operated mice underwent the same procedure, including opening of the peritoneum and exposing the bowel, but without ligation and needle perforation of the cecum. After surgery, the mice were subcutaneously injected with 1 mL of physiological saline for fluid resuscitation. Pre- and postoperatively, all mice had unlimited access to food and water.

### 2.3. Generation of Mitochondrial DNA-(mtDNA-)Deficient (*ρ*0) Cells

A549 cells were from the American Type Culture Collection (Manassas, VA, USA) and cultured in F12/DMEM medium (Invitrogen, Carlsbad, CA, USA). The medium was supplemented with 10% fetal bovine serum (FBS) and gentamicin. Cells were maintained at 37°C in humidified incubators containing an atmosphere of 95% room air and 5% CO_2_. Subconfluent cultures were used in all experiments, with the cells adhered 24 h before the experiments. A549 lung epithelial cells were grown in F12/DMEM supplemented with 10% fetal calf serum (FCS), pyruvate (100 *μ*g/mL), and uridine (50 *μ*g/mL) as described previously [32]. Ethidium bromide (EtBr, 100 ng/mL) was also added to the medium for 3 weeks. Depletion of mitochondrial DNA was evaluated by quantitative real-time PCR as described above [33]. 

### 2.4. Electron Microscopy

Cells were fixed in 2.5% glutaraldehyde in phosphate-buffered saline (PBS) after experimental manipulations. The cells were photographed using a JEM 1210 transmission electron microscope (JEOL, Peabody, MA, USA).

### 2.5. Western Blot

Western blot analysis for LD-decorating proteins and transcriptional factors was performed as previously described [29].

### 2.6. Statistics

Data are expressed as means ± SEM, and a *P* value of <0.05 was considered significant. The significance of differences between the groups was analyzed with a Student's *t*-test. Where appropriate, ANOVA with multiple comparisons followed by a Student's *t*-test was used.

## 3. Results

To gain insight into the phenotypic alteration of cells by oxygenic insults, for which we adopted several model systems.

### 3.1. Enhanced LD Formation upon Hyperoxia/Hypoxia in Mouse

**Figure 1:**
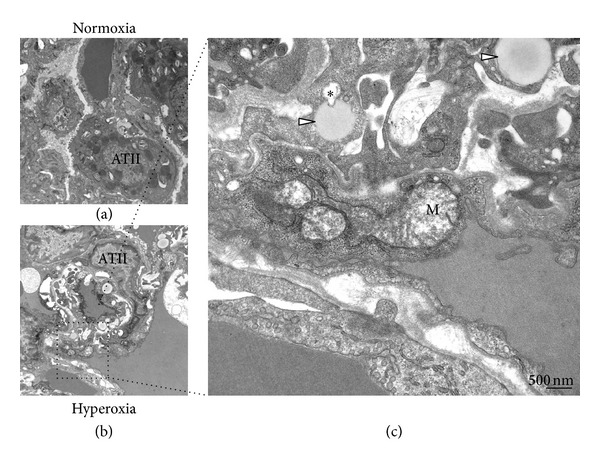
Enhanced lipid droplet (LD) formation upon hyperoxia in mouse. C57BL/6 mice were exposed to room air (a) or hyperoxia (95% O_2_, 5% N_2_) (b) for 72 h. Transmission electron microscopy (TEM) images of the lung tissue were captured. The formation of LDs (white arrowhead) in alveolar type II (ATII) cells increased upon hyperoxic exposure in the mouse lung. A select image in (b) (inset) was photographed at high power (c). *One of the LDs was invaginated by growing lysosomes as reported previously [31]. Bar = 500 nm. Approximately 50 images were captured from at least 3 individual mice of each group.

First, we examined the response of mouse lungs under hyperoxic exposure. As shown in Figure 1, the hyperoxic stimuli led to an increase in the number of LDs. Compared with endothelial cells, alveolar type II cells were more sensitive to high oxygen inhalation in terms of LD biogenesis (Figures 1(b) and 1(c)). Interestingly, endothelial mitochondria showed an enlarged morphology with swelling compared with those from air-exposed mice (Figure 1(a)).

**Figure 2:**
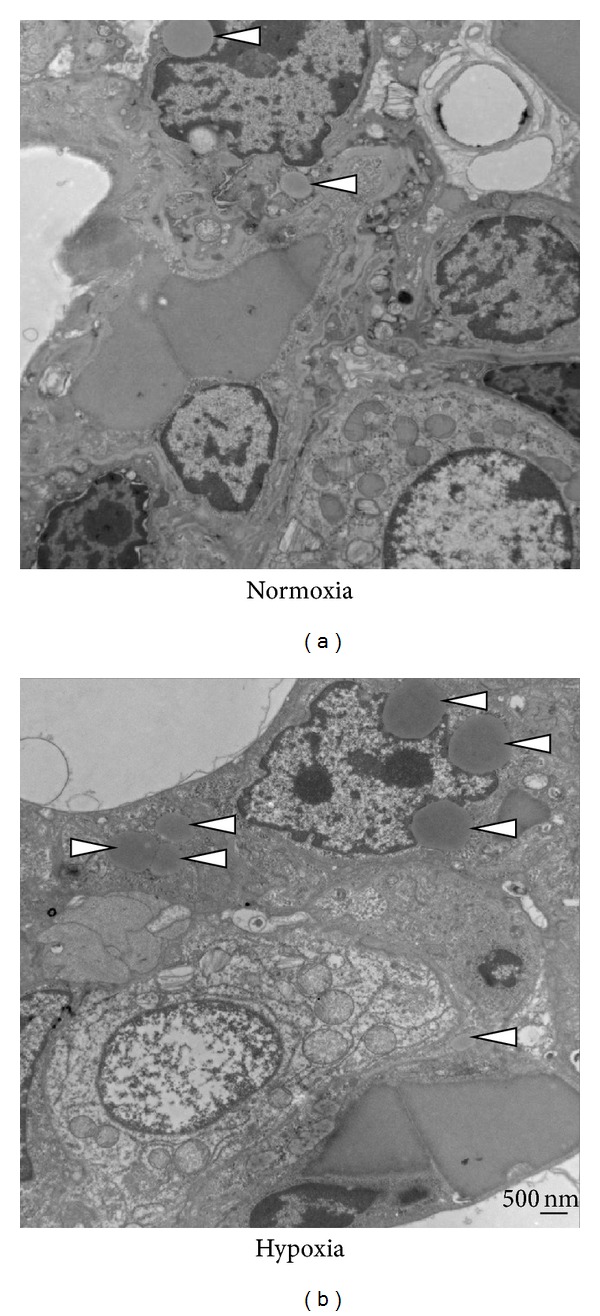
Enhanced LD formation upon hypoxia in mouse. C57BL/6 mice were exposed to room air (a) or hypoxia (10% O_2_, 5% N_2_) (b) for 3 weeks, and the lungs were harvested for further examination by TEM. The formation of LDs (white arrowhead) increased upon hypoxic exposure in the mouse lung epithelial cells. Perinuclear and cytosolic localization of LDs is shown (b). Approximately 50 images were captured from at least 3 individual mice of each group.

Chronic hypoxic exposure of mice to 10% oxygen for 3 weeks also increased the number of LDs in alveolar type II cells (Figures 2(a) and 2(b)). LDs were present close to the nucleus (Figure 2(b)) and dispersed through the cytosol as well. Under basal conditions, some LDs also localized near the nucleus (Figure 2(a)). In addition, autophagosomes were frequently observed in epithelial cells after hypoxic insult (Figure 2(b)). 

### 3.2. Myocardial Ischemia Induces LD Formation

**Figure 3:**
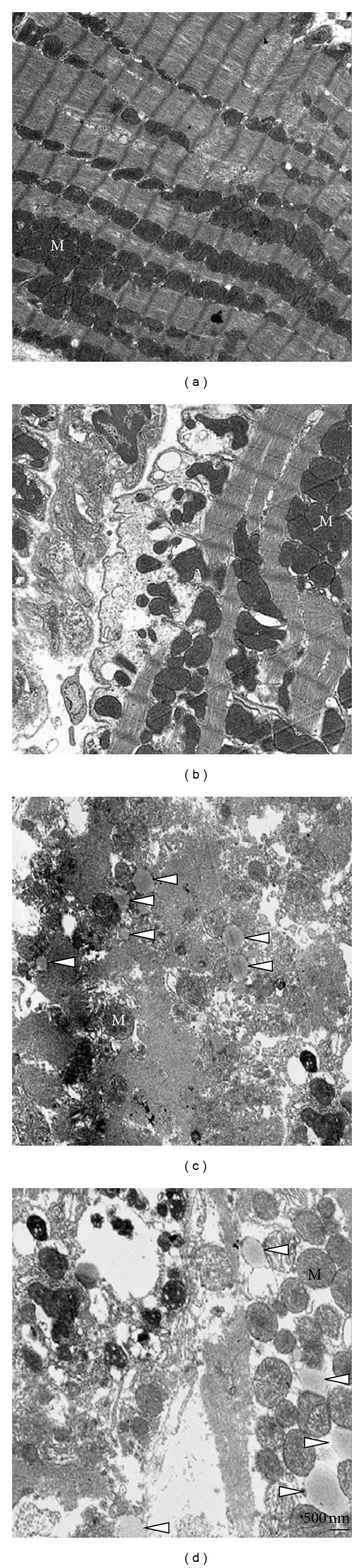
Myocardial ischemia induces LD formation. The AMI rat model was established by ligation of the left anterior descending coronary artery. The hearts were, then, harvested at 40 minutes, 24 hours after ligation in the AMI group. The rats in the sham-operated group underwent the same procedure except for the ligation of the coronary artery (a). The hearts were examined by TEM. The formation of LDs was not observed in the heart from 40-minute ligation (subinfarct, (b)) while remarked at 24-hour ligation (white arrowhead, (c) and (d)).

Under normal conditions, only a few small LDs were reported in mouse hearts [28]. In agreement with this, we found no detectible LDs under basal conditions in rat heart muscle (Figure 3(a)). Upon mild ischemic insult, mitochondrial architecture became irregularly arranged compared with air-exposed control rats (Figures 3(a) and 3(b)). The heart is unique in that it cannot fatigue while maintaining life and it must constantly generate energy in the form of ATP and phosphocreatine to perform its function. Cardiac muscle mitochondria are the major energy-producing organelles that provide working power for muscle contraction. Fatty acids are the main resources for energy production in cardiomyocytes [5]. Therefore, it is plausible that fatty acids be segregated in a safe place if the mitochondria were damaged by ischemic hypoxia. Hypoxia limits the supply of oxygen available to accept electrons in fatty acid oxidation, which resulted in mitochondrial injury, as shown in Figures 3(c) and 3(d). Newly formed LDs were mainly present near damaged mitochondria (Figure 3(d)). 

### 3.3. LD Formation in a Mouse Sepsis Model

**Figure 4:**
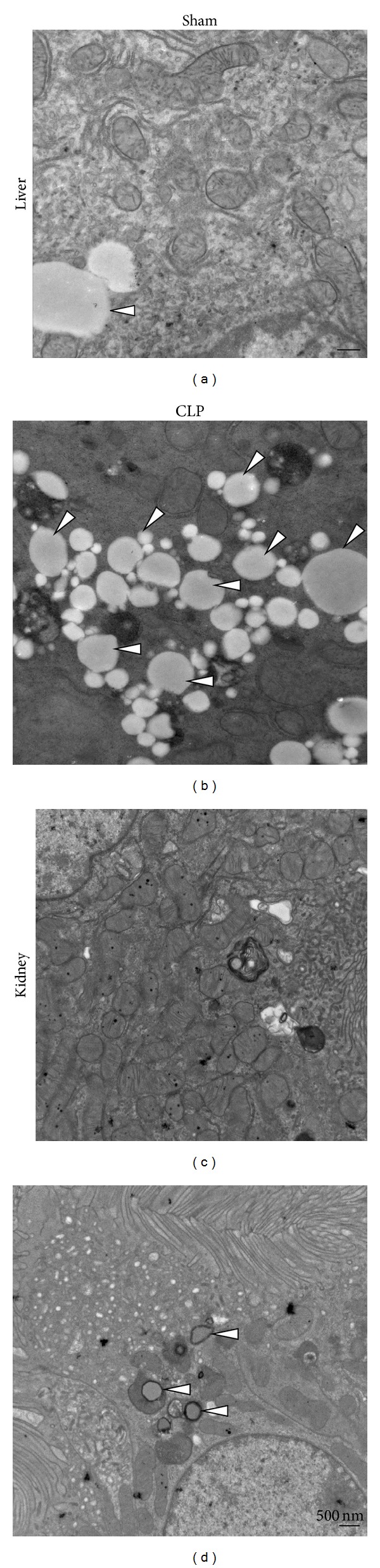
LD formation in a sepsis model in mice. Cecal ligation and puncture (CLP, 19-gauge, 1-hole) was carried out to induce polymicrobial sepsis in mice. CLP greatly enhances the formation of LDs (arrowhead) in the liver (b) compared with those in sham-operated control mice (a). To a lesser extent, the kidneys exhibited an increased number of LDs in the epithelial cells (d) compared with the control (c). Bar represents 500 nm. Approximately 50 images were captured from at least 3 individual mice of each group.

Based on recent observations, treatment of macrophages with lipopolysaccharide (LPS) has been shown to switch cellular metabolism to glycolysis rather than oxidative phosphorylation. Succinate, an intermediate of the tricarboxylic acid (TCA) cycle, accumulates relaying signals to trigger an immune reaction in those cells. In addition, increased hepatic fat storage in the form of triglycerides is a characteristic feature of sepsis [15, 16]. In agreement with this notion, our morphological data also provided evidence that CLP dramatically increased the number of LDs in the hepatocytes (Figures 4(a) and 4(b)). Modest increases in the number of LDs in the kidney tissue were observed, compared with those in the sham-operated control (Figures 4(c) and 4(d)).

### 3.4. Loss of mtDNA Increased LD Formation

**Figure 5:**
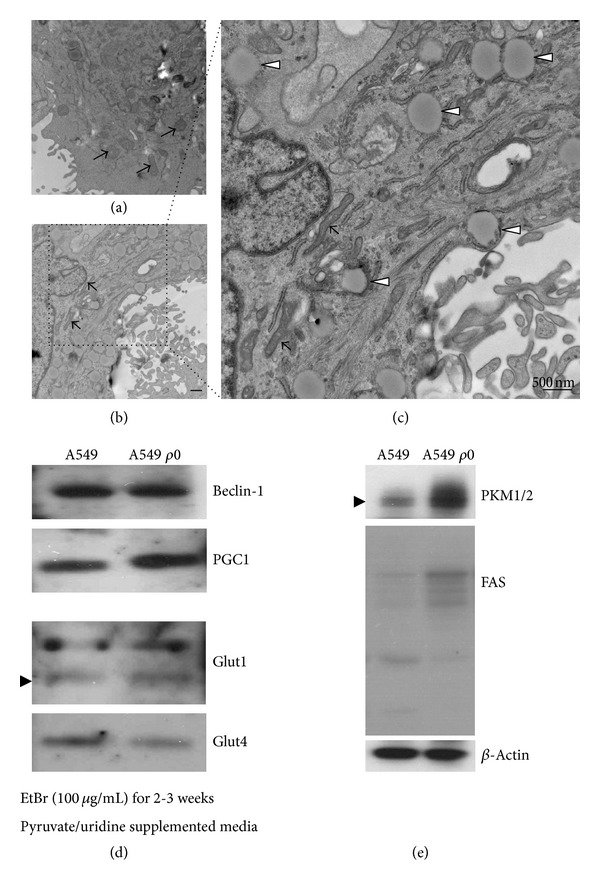
Paucity of mitochondrial DNA increased LD formation. Cells of a well-characterized human lung carcinoma cell line, A549, were treated with ethidium bromide (EtBr) for 3 weeks. Petite mitochondria were found in the cytosol of A549 *ρ*0 cells ((b), (c), arrow). A549 cells exhibit normal mitochondrial morphology with organized cristae ((a), arrow). In the vicinity of petite mitochondria, LDs developed ((c), arrowhead). Bar represents 500 nm. The expression of glucose transporters and PGC-1 was examined with cell lysates of both A549 and *ρ*0 cells (d). The expression of metabolic proteins, PKM1/2 and FAS, was also examined (e). *β*-Actin blot serves as a loading control.

It is thought that the conversion of metabolism to glycolysis is a cellular strategy against various stressors [1]. At the same time, expression of electron transport chain (ETC) proteins concomitantly diminishes. The key proteins involved in the ETC are mitochondrially translated. Hence, we mimicked this scenario by depleting mtDNA in A549 cells by treating them with low doses of EtBr for 3 weeks. The morphological changes in the mitochondria are shown in Figures 5(b) and 5(c). Most cells stopped growing. At the same time, shrunken and elongated mitochondria were ubiquitously distributed throughout the cytosol (black arrow in Figure 5(c)) of A549 *ρ*0 cells. Intriguingly, numerous LDs developed in the A549 *ρ*0 cells. The expression of PGC-1, a master transcriptional factor for mitochondria biogenesis, was augmented in the A549 *ρ*0 cells, which probably accounts for a compensatory mechanism for mtDNA loss in cells. While the expression of an insulin-sensitive glucose transporter (Glut4) decreased, that of an insulin-insensitive glucose transporter (Glut1) slightly increased (Figure 5(d)). To measure metabolic protein expression, we examined pyruvate kinase M2 (PKM2). PKM2 favors glycolysis over oxidative phosphorylation for glucose oxidation. PKM2 expression was upregulated in A549 *ρ*0 cells. Moreover, the expression of fatty acid synthase (FAS) was significantly increased in A549 *ρ*0 cells (Figure 5(e)), suggesting the development of LDs in response to the shortage of mtDNA. 

### 3.5. Limited LD Formation in the Absence of HO-1 Activity

**Figure 6:**
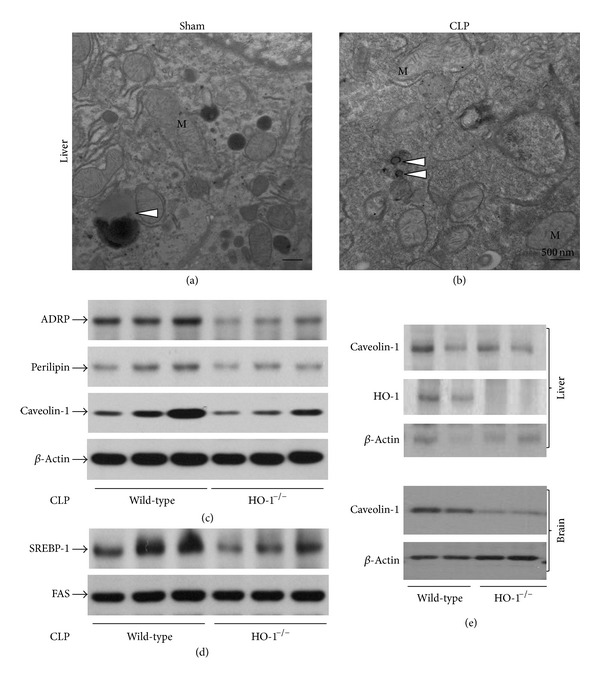
A defect in hepatic LD formation by CLP in heme oxygenase (HO)-1-deficient mouse. Polymicrobial sepsis was induced as described in Figure 4 in HO-1-null and control mice. Very few LDs were observed in both wild-type (a) and HO-1-null mice (b). The expression of LD coat proteins and caveolin-1 was examined (c). FAS levels are generally unaltered in the absence of HO-1 (d). Measurement of basal levels of caveolin-1 and validation of HO-1-knockout mice were carried out with both cell lysates (e). *β*-Actin blot serves as a loading control.

Because oxygenic stress and inflammation induce HO-1 and mitochondrial dysfunction triggers LD formation, we hypothesized that HO-1 activity mediates LD formation in response to mitochondrial defects. As opposed to LD formation in CLP livers (Figure 4(b)), fewer LDs were found in the liver of HO-1-deficient mice (Figure 6(b)). Without CLP, there is no difference in the number of LDs irrespective of the presence of HO-1 (Figures 4(a) and 6(a)). In accordance with LD formation in sepsis, the expression of the LD-decorating proteins, ADRP and perilipin, was increased compared with HO-1-null livers (Figure 6(c)). Caveolin-1 expression was also upregulated. Caveolin-1 is a recently described protein that participates in LD formation and lipid metabolism. PPAR-*γ*2 modulates LD biogenesis through SREBP-1 and FAS (Figure 5(e)). The expression of SREBP-1 was robust in wild-type liver but not in HO-1-null liver (Figure 6(d)). We validated HO-1 expression in HO-1-deficient liver (Figure 6(e)). Basal expression of caveolin-1 was downregulated in the brain of HO-1-null mice, but it was slightly increased in the liver (Figure 6(e)). Lipid accumulation and the levels of the nuclear form of SREBP, in particular subtype 1c, were positively correlated in hydrogen peroxide-injured hepatocytes. Thus, it has been postulated that ROS may stimulate LD formation via SREBP-1 activation [34].

## 4. Discussion

In this study, we examined LD formation as a general response to various stresses *in vivo* and *in vitro*. Ample evidence suggests that in response to oxidative stress [35], organisms can drive their metabolic flux from oxidative phosphorylation to glycolysis, including the pentose phosphate pathway [36], which provides the reducing power for the maintenance of cellular redox potential. LD biogenesis during stress can trigger adaptive LD formation, which depends on mitochondrial integrity and HO-1 activity; this may be a cellular survival strategy, apportioning energy-generating substrates toward cellular defense.

Storage of fatty acids is common to all three domains of life, including bacteria, archaea, and eukaryotes [37, 38]. The specialized organelles for fat storage are called lipid bodies, lipid inclusions, or LDs. These organelles share essentially the same phenotype, comprising a hydrophobic core of lipids surrounded by a monolayer of phospholipids. Therefore, LDs in both worms and mammals, for example, are regarded as evolutionarily conserved structures that store fatty acids. Although LD accumulation in fat tissues was once considered notorious for the obesity-related disorders, LD formation is actually cytoprotective, since failure to sequester fat into LDs can cause cellular stress and insulin resistance [5, 34, 37].

Upon exogenous oxygenic insults, organisms protect themselves by homeostatic modulation of reactive oxygen species (ROS). One significant source of ROS is generated by lipid *β*-oxidation. FADH_2_ generated during the *β*-oxidation of fatty acids enhances the production of ROS via reversing the electron flow from electron transport chain complex II to complex I, a major source of ROS in the mitochondria. Therefore, the sequestration of fatty acids within LDs, preventing *β*-oxidation, is a plausible survival strategy and mechanism to protect cellular integrity against stress [34]. 

Insulin reflects an energy surplus, pushing cells to store the energy as fat and glycogen in insulin-sensitive organs [39, 40]. At the organism level, however, “transient” insulin resistance is assumed to be critical in various states such as starvation, infection, trauma, and even cancer [41–44] to spare glucose for different biosynthetic purposes, such as the production of NADPH nucleotides via the pentose phosphate pathway [36, 40]. Under these conditions, total glucose oxidation by the TCA cycle is actually low, and energy demands are primarily met by fatty acid and ketone body oxidation [40]. In our current study, we aimed to understand LD formation in response to oxygenic stress and sepsis and to further reveal the underlying mechanism in mitochondria. 

The importance of LDs is nicely exemplified in a liver regeneration model following partial hepatectomy in mice. Liver regeneration is an orchestrated cellular response that coordinates lipid metabolism and cell division. Fernàndez et al. and the Lisanti group demonstrated that caveolin-1-deficient mice exhibit impaired liver regeneration [17, 19], thereby resulting in a low survival rate after partial hepatectomy. Reduced LD accumulation in the hepatocytes of caveolin-1-null mice was responsible for blockade of the cell cycle, since the infusion of glucose counteracts and reverses growth arrest. Glucose administration would fulfill the energetic and biosynthetic intermediate requirements. This is evidenced by a transient increase in the liver triacylglycerol levels in glucose-fed animals. Therefore, distribution and allotment of available resources, such as LD biogenesis, may be a critical factor for wound healing and resistance against stress. This also implies that LD formation has a protective role against lipotoxicity derived from free fatty acid accumulation inside cells. These fatty acids can then be used for structural functions and additional cellular requirements. 

Surprisingly, injured mitochondria are involved in the formation of LDs under stressed conditions. As expected, depletion of mtDNA causes morphological changes and alteration of metabolic protein expression. The glucose transport proteins facilitate glucose transport into insulin-sensitive cells [39]. Glut1 is insulin independent and is ubiquitously distributed in different tissues. Glut4 is insulin dependent and is responsible for the majority of glucose transport into muscle and adipose cells under anabolic conditions. Park et al. reported that loss of mitochondrial DNA by chronic treatment with EtBr downregulates glucose transporters and reduces intracellular ATP levels in hepatoma cells [45]. They measured protein expression of glucose transporters and found reduced expression of Glut4. In agreement with this, A549 *ρ*0 cells showed diminished expression of Glut4 (Figure 5(d)), while the expression of Glut1 was slightly increased (Figure 5(d)). In addition, glycolysis and fatty acid synthesis may be augmented as assessed by PKM2 and FAS upregulation (Figure 5(e)). Our results suggest that mtDNA loss results in metabolic changes resembling the “transient” insulin-resistant phenotype. 

High-yield extraction of ATP from glucose can be obtained by oxidative phosphorylation [46], but nothing remains for use as cellular building blocks. Therefore, oxidative phosphorylation is suitable for normal processes in fully differentiated cells such as neuronal cells. If cells must cope with adverse situations such as oxidative stress or bacterial infection [36, 38], the strategy needs to be altered to spare nutrients for establishing cellular redox status and recovering damaged cell structures. Glycolysis can be branched out to the oxidative pentose phosphate pathway, which is a major source of both reducing power and metabolic intermediates for biosynthetic processes in cells.

All life on Earth is able to sense oxygen levels in the environment. Hypoxia, for example, can be managed by stabilization of hypoxia-inducible factor- (HIF-)1*α*. HIF-1*α* modulates angiogenesis at the organism level [30] but switches glucose catabolism to glycolysis at the cellular level. Both functions are considered homeostatic adjustments to oxygen. A recent report suggested that hypoxia-inducible protein 2 (HIG2), which is a downstream target of HIF-1*α* transcriptional activity, is implicated in LD biogenesis [35]. HIG2 localizes to LD membranes and coexists with ADRP and perilipin. Overexpression of HIG2 is sufficient to increase in LD accumulation under normal conditions. Our data also support the idea of accumulation of fatty acids in LDs upon hypoxia and hyperoxia (Figures 1–3). 

If oxidative stress induces mitochondrial dysfunction, the organelle sends signals to the nucleus for the recovery of mitochondria and cellular integrity [47, 48]. We previously demonstrated that HO-1 activity might be mediated by the retrograde signal transduction originating from endoplasmic stresses [23, 26]. In this study, we also have shown that without HO-1 activity, LD formation in septic liver tissue is greatly attenuated (Figure 6). In the absence of HO-1 activity, mice were vulnerable to bacterial infection by CLP, with increased mortality rate [21]. The role of LDs in the protective activity of HO-1 in the CLP model and in response to other stressors warrants further investigation. 

Glycolysis is upregulated in some cancer cells. Such shifts toward a glycolytic phenotype have been explored in other physiological situations. The molecular mechanisms underlying these shifts have been focused on the glycolytic enzymes such as muscle-type phosphofructokinase or pyruvate kinase M. On the other hand, mitochondrial inhibition of ATP synthesis could also act as a trigger for the initiation of glycolysis via ATPase inhibitory factor- (IF-)1. Thus, multiple pathways are operating to switch cellular metabolism under stressed conditions. ATP production appears to be significantly reduced by the inhibition of heme biosynthesis with succinylacetone (SA), since ATPase IF-1 expression was remarkably augmented by SA (data not shown). Diminished ATP production resulted in mitochondrial dysfunction and switching to glycolysis for glucose catabolism [46]. Heme proteins that mainly operate in the ETC would be degraded by the heme catabolic enzyme HO-1. In this context, it has been reported that carbon monoxide, a byproduct of HO-1 activity, relays retrograde signals in neuronal systems [48]. Therefore, the role of HO-1 activity in linking mitochondrial dysfunction to LD biogenesis warrants further study. 

Collectively, mitochondrial dysfunction may trigger LD formation by switching metabolic pathways to glycolysis and fatty acid biosynthesis. During this process, HO-1 activity appears necessary to form LDs through upregulation of proteins for LD trafficking and lipid metabolism. 

## References

[1] Greenberg AS, Coleman RA, Kraemer FB (2011). The role of lipid droplets in metabolic disease in rodents and humans. *Journal of Clinical Investigation*.

[2] MacPherson REK, Herbst EAF, Reynolds EJ, Vandenboom R, Roy BD, Peters SJ (2012). Subcellular localization of skeletal muscle lipid droplets and PLIN family proteins OXPAT and ADRP at rest and following contraction in rat soleus muscle. *The American Journal of Physiology—Regulatory Integrative and Comparative Physiology*.

[3] Storey SM, McIntosh AL, Senthivinayagam S, Moon KC, Atshaves BP (2011). The phospholipid monolayer associated with perilipin-enriched lipid droplets is a highly organized rigid membrane structure. *The American Journal of Physiology—Endocrinology and Metabolism*.

[4] Torday J, Rehan V (2011). Neutral lipid trafficking regulates alveolar type II cell surfactant phospholipid and surfactant protein expression. *Experimental Lung Research*.

[5] Meex RCR, Schrauwen P, Hesselink MKC (2009). Modulation of myocellular fat stores: lipid droplet dynamics in health and disease. *The American Journal of Physiology—Regulatory Integrative and Comparative Physiology*.

[6] Olofsson SO, Boström P, Andersson L, Rutberg M, Perman J, Borén J (2009). Lipid droplets as dynamic organelles connecting storage and efflux of lipids. *Biochimica et Biophysica Acta*.

[7] Kim HJ, Jung TW, Kang ES (2007). Depot-specific regulation of perilipin by rosiglitazone in a diabetic animal model. *Metabolism*.

[8] Boehme DH, Sobel HJ, Marquet E, Salen G (1980). Liver in Cerebrotendinous xanthomatosis (CTX). A histochemical and EM study of four cases. *Pathology Research and Practice*.

[9] Sørensen TIA, Virtue S, Vidal-Puig A (2010). Obesity as a clinical and public health problem: is there a need for a new definition based on lipotoxicity effects?. *Biochimica et Biophysica Acta*.

[10] Paul A, Chan L, Bickel PE (2008). The PAT family of lipid droplet proteins in heart and vascular cells. *Current Hypertension Reports*.

[11] Olofsson SO, Boström P, Andersson L (2008). Triglyceride containing lipid droplets and lipid droplet-associated proteins. *Current Opinion in Lipidology*.

[12] Miranda DA, Koves TR, Gross DA (2011). Re-patterning of skeletal muscle energy metabolism by fat storage-inducing transmembrane protein 2. *The Journal of Biological Chemistry*.

[13] Ivashov VA, Zellnig G, Grillitsch K, Daum G (2013). Identification of triacylglycerol and steryl ester synthases of the methylotrophic yeast *Pichia pastoris*. *Biochimica et Biophysica Acta*.

[14] Ueno M, Suzuki J, Zenimaru Y (2008). Cardiac overexpression of hormone-sensitive lipase inhibits myocardial steatosis and fibrosis in streptozotocin diabetic mice. *The American Journal of Physiology—Endocrinology and Metabolism*.

[15] Khatchadourian A, Bourque SD, Richard VR, Titorenko VI, Maysinger D (2012). Dynamics and regulation of lipid droplet formation in lipopolysaccharide (LPS)-stimulated microglia. *Biochimica et Biophysica Acta*.

[16] Younce C, Kolattukudy P (2012). MCP-1 induced protein promotes adipogenesis via oxidative stress, endoplasmic reticulum stress and autophagy. *Cellular Physiology and Biochemistry*.

[17] Fernàndez MA, Albor C, Ingelmo-Torres M (2006). Caveolin-1 is essential for liver regeneration. *Science*.

[18] Blouin CM, Le Lay S, Eberl A (2010). Lipid droplet analysis in caveolin-deficient adipocytes: alterations in surface phospholipid composition and maturation defects. *Journal of Lipid Research*.

[19] Cohen AW, Razani B, Schubert W (2004). Role of caveolin-1 in the modulation of lipolysis and lipid droplet formation. *Diabetes*.

[20] Fernández-Rojo MA, Restall C, Ferguson C (2012). Caveolin-1 orchestrates the balance between glucose and lipid-dependent energy metabolism: implications for liver regeneration. *Hepatology*.

[21] Su WC, Liu X, Macias AA, Baron RM, Perrella MA (2008). Heme oxygenase-1-derived carbon monoxide enhances the host defense response to microbial sepsis in mice. *Journal of Clinical Investigation*.

[22] Piantadosi CA, Withers CM, Bartz RR (2011). Heme oxygenase-1 couples activation of mitochondrial biogenesis to anti-inflammatory cytokine expression. *The Journal of Biological Chemistry*.

[23] Slebos D, Ryter SW, van der Toorn M (2007). Mitochondrial localization and function of heme oxygenase-1 in cigarette smoke-induced cell death. *The American Journal of Respiratory Cell and Molecular Biology*.

[24] Piantadosi CA, Carraway MS, Babiker A, Suliman HB (2008). Heme oxygenase-1 regulates cardiac mitochondrial biogenesis via nrf2-mediated transcriptional control of nuclear respiratory factor-1. *Circulation Research*.

[25] Kim HP, Ryter SW, Choi AM (2006). CO as a signaling molecule. *Annual Review of Pharmacology and Toxicology*.

[26] Ryter SW, Alam J, Choi AMK (2006). Heme oxygenase-1/carbon monoxide: from basic science to therapeutic applications. *Physiological Reviews*.

[27] Tanaka A, Jin Y, Lee SJ (2012). Hyperoxia-induced LC3B interacts with the Fas apoptotic pathway in epithelial cell death. *The American Journal of Respiratory Cell and Molecular Biology*.

[28] Lee SJ, Smith A, Guo L (2011). Autophagic protein LC3B confers resistance against hypoxia-induced pulmonary hypertension. *The American Journal of Respiratory and Critical Care Medicine*.

[29] Lee SJ, Kim HP, Jin Y, Choi AMK, Ryter SW (2011). Beclin 1 deficiency is associated with increased hypoxia-induced angiogenesis. *Autophagy*.

[30] Zhang J, Lu J, Chen D (2009). Myocardial autophagy variation during acute myocardial infarction in rats: the effects of carvedilol. *Chinese Medical Journal*.

[31] Singh R, Kaushik S, Wang Y (2009). Autophagy regulates lipid metabolism. *Nature*.

[32] Bodnar AG, Cooper JM, Holt IJ, Leonard JV, Schapira AHV (1993). Nuclear complementation restores mtDNA levels in cultured cells from a patient with mtDNA depletion. *The American Journal of Human Genetics*.

[33] Nakahira K, Haspel JA, Rathinam VAK (2011). Autophagy proteins regulate innate immune responses by inhibiting the release of mitochondrial DNA mediated by the NALP3 inflammasome. *Nature Immunology*.

[34] Sekiya M, Hiraishi A, Touyama M, Sakamoto K (2008). Oxidative stress induced lipid accumulation via SREBP1c activation in HepG2 cells. *Biochemical and Biophysical Research Communications*.

[35] Gimm T, Wiese M, Teschemacher B (2010). Hypoxia-inducible protein 2 is a novel lipid droplet protein and a specific target gene of hypoxia-inducible factor-1. *FASEB Journal*.

[36] Perl A, Hanczko R, Telarico T, Oaks Z, Landas S (2011). Oxidative stress, inflammation and carcinogenesis are controlled through the pentose phosphate pathway by transaldolase. *Trends in Molecular Medicine*.

[37] Soeters MR, Soeters PB (2012). The evolutionary benefit of insulin resistance. *Clinical Nutrition*.

[38] Anand P, Cermelli S, Li Z (2012). A novel role for lipid droplets in the organismal antibacterial response. *Elife*.

[39] Ebeling P, Koistinen HA, Koivisto VA (1998). Insulin-independent glucose transport regulates insulin sensitivity. *FEBS Letters*.

[40] van Cromphaut SJ, Vanhorebeek I, van den Berghe G (2008). Glucose metabolism and insulin resistance in sepsis. *Current Pharmaceutical Design*.

[41] Frank P, Katz A, Andersson E, Sahlin K (2013). Acute exercise reverses starvation-mediated insulin resistance in humans. *The American Journal of Physiology—Endocrinology and Metabolism*.

[42] Lindegaard B, Hvid T, Grøndahl T (2013). Expression of fibroblast growth factor-21 in muscle is associated with lipodystrophy, insulin resistance and lipid disturbances in patients with HIV. *PLoS ONE*.

[43] Bonizzoli M, Zagli G, Lazzeri C, Degl'Innocenti S, Gensini G, Peris A (2012). Early insulin resistance in severe trauma without head injury as outcome predictor? A prospective, monocentric pilot study. *Scandinavian Journal of Trauma, Resuscitation and Emergency Medicine*.

[44] Capasso I, Esposito E, Pentimalli F (2013). Homeostasis model assessment to detect insulin resistance and identify patients at high risk of breast cancer development: national cancer institute of naples experience. *Journal of Experimental and Clinical Cancer Research*.

[45] Park KS, Nam KJ, Kim JW (2001). Depletion of mitochondrial DNA alters glucose metabolism in SK-Hep1 cells. *The American Journal of Physiology—Endocrinology and Metabolism*.

[46] Formentini L, Sánchez-Aragó M, Sánchez-Cenizo L, Cuezva JMC (2012). The mitochondrial ATPase inhibitory factor 1 triggers a ROS-mediated retrograde prosurvival and proliferative response. *Molecular Cell*.

[47] Bonnard C, Durand A, Peyrol S (2008). Mitochondrial dysfunction results from oxidative stress in the skeletal muscle of diet-induced insulin-resistant mice. *Journal of Clinical Investigation*.

[48] Lancel S, Hassoun SM, Favory R, Decoster B, Motterlini R, Neviere R (2009). Carbon monoxide rescues mice from lethal sepsis by supporting mitochondrial energetic metabolism and activating mitochondrial biogenesis. *Journal of Pharmacology and Experimental Therapeutics*.

